# The Discovery of Penicillin—New Insights After More Than 75 Years of Clinical Use

**DOI:** 10.3201/eid2305.161556

**Published:** 2017-05

**Authors:** Robert Gaynes

**Affiliations:** Emory University School of Medicine, Decatur, Georgia, USA

**Keywords:** penicillin, discovery, antimicrobial drugs, antibiotics, history of medicine

## Abstract

After just over 75 years of penicillin’s clinical use, the world can see that its impact was immediate and profound. In 1928, a chance event in Alexander Fleming’s London laboratory changed the course of medicine. However, the purification and first clinical use of penicillin would take more than a decade. Unprecedented United States/Great Britain cooperation to produce penicillin was incredibly successful by 1943. This success overshadowed efforts to produce penicillin during World War II in Europe, particularly in the Netherlands. Information about these efforts, available only in the last 10–15 years, provides new insights into the story of the first antibiotic. Researchers in the Netherlands produced penicillin using their own production methods and marketed it in 1946, which eventually increased the penicillin supply and decreased the price. The unusual serendipity involved in the discovery of penicillin demonstrates the difficulties in finding new antibiotics and should remind health professionals to expertly manage these extraordinary medicines.

 According to British hematologist and biographer Gwyn Macfarlane, the discovery of penicillin was “a series of chance events of almost unbelievable improbability” (*1*). After just over 75 years of clinical use, it is clear that penicillin’s initial impact was immediate and profound. Its detection completely changed the process of drug discovery, its large-scale production transformed the pharmaceutical industry, and its clinical use changed forever the therapy for infectious diseases. The success of penicillin production in Great Britain and the United States overshadowed the serendipity of its production and the efforts of other nations to produce it. Information on penicillin production in Europe during World War II, available only in the last 10–15 years, provides new insights into penicillin’s story.

## Dawn of Chemotherapy and the “Magic Bullet”

At the beginning of the 20th century, Paul Ehrlich pioneered the search for a chemical that would kill a microorganism and leave the host unaltered—the “magic bullet.” Ehrlich also coined the term chemotherapy: “There must be planned chemical synthesis: proceeding from a chemical substance with recognizable activity, making derivatives from it, and then trying each to discover the degree of its activity and effectiveness. This we call chemotherapy” (*2*). After extensive testing, he found a drug with activity against the bacterium *Treponema pallidum*, which causes syphilis. The introduction of this drug, arsphenamine (Salvarsan), and its chemical derivative neoarsphenamine (Neosalvarsan) in 1910 ushered in a complete transformation of syphilis therapy and the concept of chemotherapy. Unfortunately, despite exhaustive searches, the promise of more magic bullets for microbial therapy remained elusive. For 20 years, Salvarsan and Neosalvarsan were the only chemotherapy for bacterial infections.

## Alexander Fleming’s Discovery 

A chance event in a London laboratory in 1928 changed the course of medicine. Alexander Fleming, a bacteriologist at St. Mary’s Hospital, had returned from a vacation when, while talking to a colleague, he noticed a zone around an invading fungus on an agar plate in which the bacteria did not grow. After isolating the mold and identifying it as belonging to the *Penicillium* genus, Fleming obtained an extract from the mold, naming its active agent penicillin. He determined that penicillin had an antibacterial effect on staphylococci and other gram-positive pathogens.

Fleming published his findings in 1929 (*3*). However, his efforts to purify the unstable compound from the extract proved beyond his capabilities. For a decade, no progress was made in isolating penicillin as a therapeutic compound. During that time, Fleming sent his *Penicillium* mold to anyone who requested it in hopes that they might isolate penicillin for clinical use. But by the early 1930s, interest had waned in bringing to life Paul Ehrlich’s vision of finding the magic bullet.

## Discovery of Prontosil and Sulfa Drugs

This dismal outlook on chemotherapy began to change when Gerhard Domagk, a German pathologist and bacteriologist, found bacteriologic activity in a chemical derivative from oil dyes called sulfamidochrysoïdine (also known as Prontosil). This compound had bacteriologic activity in animals, but strangely, none in vitro*.* Prontosil had limited but definite success when used to treat patients with bacterial infections, including Domagk’s own child. A German company patented the drug, and ultimately, Domagk won a Nobel Prize in 1939. The paradox of Prontosil’s in vivo success but lack of success in vitro was explained in 1935, when French scientists determined that only part of Prontosil was active: sulfanilamide. In animals, Prontosil was metabolized into sulfanilamide. Within 2 years, sulfanilamide and several derivative sulfa drugs were on the market. The success of sulfanilamide changed the cynicism about chemotherapy of bacteria (*1*).

## Isolation of Penicillin at Oxford University

The success of sulfa drugs sparked interest in finding other agents. At Oxford University, Ernst Chain found Fleming’s 1929 article on penicillin and proposed to his supervisor, Howard Florey, that he try to isolate the compound. Florey’s predecessor, George Dreyer, had written Fleming earlier in the 1930s for a sample of his strain of *Penicillium* to test it for bacteriophages as a possible reason for antibacterial activity (it had none). However, the strain had been saved at Oxford. In 1939, Howard Florey assembled a team, including a fungal expert, Norman Heatley, who worked on growing *Penicillium* spp. in large amounts, and Chain, who successfully purified penicillin from an extract from the mold. Florey oversaw the animal experiments. On May 25, 1939, the group injected 8 mice with a virulent strain of *Streptococcus* and then injected 4 of them with penicillin; the other 4 mice were kept as untreated controls. Early the next morning, all control mice were dead; all treated mice were still alive. Chain called the results “a miracle.” The researchers published their findings in The Lancet in August 1940, describing the production, purification, and experimental use of penicillin that had sufficient potency to protect animals infected with *Streptococcus pyogenes, Staphylococcus aureus,* and *Clostridium septique* (*4*).

After the Oxford team had purified enough penicillin, they began to test its clinical effectiveness. In February 1941, the first person to receive penicillin was an Oxford policeman who was exhibiting a serious infection with abscesses throughout his body. The administration of penicillin resulted in a startling improvement in his condition after 24 hours. The meager supply ran out before the policeman could be fully treated, however, and he died a few weeks later. Other patients received the drug with great success. The Oxford team then published their clinical findings (*5*). At the time, however, pharmaceutical companies in Great Britain were unable to mass produce penicillin because of World War II commitments. Florey then turned to the United States for assistance.

## Penicillin and US Involvement

In June 1941, Florey and Heatley traveled to the United States. Concerned about the security of taking a culture of the precious *Penicillium* mold in a vial that could be stolen, Heatley suggested that they smear their coats with the *Penicillium* strain for safety on their journey. They eventually arrived in Peoria, Illinois, to meet with Charles Thom, the principal mycologist of the US Department of Agriculture, and Andrew Jackson Moyer, director of the department’s Northern Research Laboratory. Thom corrected the identification of Fleming’s mold to *P. notatum*; it was initially identified as *P. rubrum* (*1*).

Thom also recognized the rarity of this *P. notatum* strain because only 1 other strain in his collection of 1,000 *Penicillium* strains produced penicillin. The strain that was eventually used in mass production was a third strain, *P. chrysogenum*, found in a moldy cantaloupe in a market, which produced 6 times more penicillin than Fleming’s strain. When a component of the media that Heatley used to grow the mold in England was unavailable, A.J. Moyer suggested using corn steep liquor, a waste product from the manufacture of cornstarch that was available in large quantities in the midwestern United States. With corn steep liquor, the investigators produced exponentially greater amounts of penicillin in the filtrate of the mold than the Oxford team had ever produced. Heatley remained in Peoria for 6 months to work on methods of growing *Penicillium* strains in large quantities. Florey headed east to interest the US government and multiple drug companies in penicillin production. The US government took over all penicillin production when the United States entered World War II. Researchers at drug companies developed a new technique for producing enormous quantities of penicillin-producing *Penicillium* spp*.*: deep-tank fermentation. This process adapted a fermentation process performed in swallow dishes to deep tanks by bubbling air through the tank while agitating it with an electric stirrer to aerate and stimulate the growth of tremendous quantities of the mold. Unprecedented United States/Great Britain cooperation for penicillin production was incredibly successful. In 1941 the United States did not have sufficient stock of penicillin to treat a single patient. At the end of 1942, enough penicillin was available to treat fewer than 100 patients. By September 1943, however, the stock was sufficient to satisfy the demands of the Allied Armed Forces (*6*).

## Public Awareness: The Fleming Myth

Early in 1942, Florey and Heatley went back to England. Because of the shortage of penicillin supplies coming from the United States, the Oxford group still had to produce most of the penicillin they tested and used. In August 1942, Fleming obtained some of the Oxford group’s supply and successfully treated a patient who was dying of streptococcal meningitis. When the patient recovered, the cure was the subject of a major article in The Times newspaper in Great Britain, which named Oxford as the source of the penicillin. However, neither Florey nor Fleming was acknowledged in the article, an oversight quickly corrected by Fleming’s boss, Sir Almroth Wright. He wrote a letter to The Times expounding on Fleming’s work and suggested that Fleming deserved a “laurel wreath.” Fleming happily talked to the press. Florey not only did not speak with the press but prohibited any member of the Oxford team from giving interviews, leading many to erroneously believe that Fleming alone was responsible for penicillin.

## Secrecy in Wartime England

The British government went to great lengths to prevent the means for producing penicillin from falling into enemy hands. However, news about penicillin leaked out. A Swiss company (CIBA, Basal, Switzerland) wrote to Florey requesting *P. notatum*. Concerned about responding, Florey contacted the British government. Agents attempted to track down where Fleming’s *Penicillium* cultures had been distributed. Fleming wrote, “During the past 10 years I have sent out a very large number of cultures of *Penicillium* to all sorts of places, but as far as I can remember NONE have gone to Germany” (*7*). Florey believed that, without the mold, no one in Germany could produce penicillin even though his publication had provided a “blueprint” for its small scale manufacture. Florey was wrong, and so was Fleming.

Fleming had sent a culture of *Penicillium* strains to “Dr. H. Schmidt” in Germany in the 1930s. Schmidt was unable to get strain to grow, but even though the Germans did not have a viable strain, other Europeans did.

## Production during World War II

### France

Someone at Institut Pasteur in France, had Fleming’s strain. In 1942, efforts began at Institut Pasteur and Rhone-Poulenc to produce penicillin. Eventually, German officials found out and, in early 1944, the Germans asked the French for their *P. notatum*. They were given a false strain that did not produce penicillin. With limited supplies, the French produced only enough penicillin to treat ≈30 patients before the war’s end.

### The Netherlands 

The situation in the Netherlands was different. The Centraalbureau voor Schimmelcultures (CBS) near Utrecht had the largest fungal collection in the world. A published list of their strains in 1937 included *P. notatum*. A letter found at CBS shows that in February 1942 the Nazis asked CBS to send their strain of *P. notatum* to Dr. Schmidt in Germany, mentioning penicillin in the letter. CBS told the Germans they did not have Fleming’s strain of *P. notatum*. In fact, they did. In the 1930s, Fleming had sent his strain to Johanna Westerdijk, the CBS director. Westerdijk could not refuse the German request for their strain of *P. notatum* but sent them the one that did not produce penicillin.

Efforts to produce penicillin in the Netherlands went underground at a company in Delft, the Nederladsche Gist-en Spiritusfabriek (the Netherlands Yeast and Spirit Factory, NG&SF). After the German occupation in 1940, NG&SF was still allowed to function. Because Delft was not bombed in the war, NG&SF’s efforts were unaffected. In early 1943, NG&SF’s executive officer, F.G. Waller, secretly wrote to Westerdijk at CBS, asking for any *Penicillium* strains that produced penicillin. In January 1944, Westerdijk sent all of CBS’ *Penicillium* strains to NG&SF.

Four reports in NG&SF records detailed their efforts (*8*). In the first report, NG&SF scientists tested 18 *Penicillium* strains from CBS; they found 1 strain with the greatest antibacterial activity, which was coded P-6 and was identified as *P. baculatum.* The second report discussed how NG&SF scientists then isolated an extract from P-6. They gave the substance in the extract the code name Bacinol after the species from which it was derived and to keep the Germans unaware of what they were doing (Figure). As Waller wrote, “When we first started looking, in 1943, only one publication was available, that of Fleming in 1929. It was on that basis we started our research” (*6*). NG&SF researchers then had help from an unanticipated source. In 1939, Andries Querido was employed by NG&SF as a part-time advisor. By January 1943, however, his Jewish background limited his visits. On his last visit in the summer of 1944, Querido met someone in Amsterdam‘s Central Train Station who gave him a copy of the latest Schweizerische Medizinische Wochenschrift (Swiss Medical Journal), which he passed on to the NG&SF scientists. The June 1944 issue contained an article entirely devoted to penicillin, showing the results that the Allies had achieved, including details of penicillin growth in corn steep extract, the scaling up of penicillin production, the measurement of strength by the Oxford unit, results of animal and human studies, and identification of the bacteria known to be susceptible to penicillin. The third report described how NG&SF scientists isolated Bacinol from the extract using the information supplied secretly by Querido.

**Figure:**
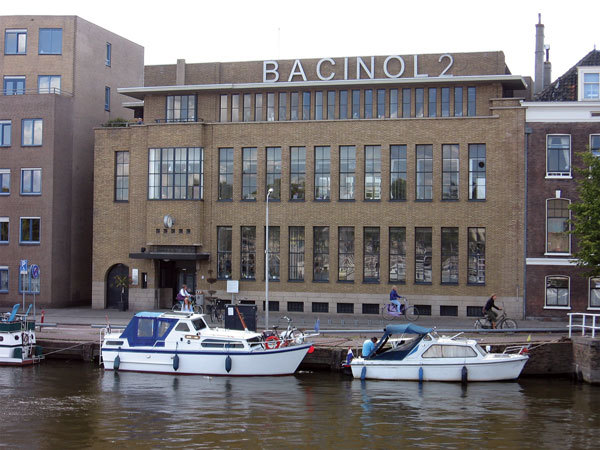
Bacinol 2, building named in honor of the site of efforts in the Netherlands to produce penicillin during World War II and the drug produced by the Netherlands Yeast and Spirit Factory in Delft. Bacinol was a code name for penicillin. Source: https://commons.wikimedia.org/wiki/File:Delft_-_Gevel_Bacinol_2.jpg

Large-scale production would be difficult to do and to keep secret from the Germans, especially with a German guard on site. However, NG&SF scientists used an obvious ploy to keep the German guard, who knew nothing about microbiology, at bay: they kept him drunk. “We did have a German guard whose job it was to keep us under surveillance, but he liked gin, so we made sure he got a lot. He slept most afternoons” (*6*). NG&SF scientists used milk bottles for growing large quantities of *Penicillium* mold. From July 1944 until March 1945, production of Bacinol continued, as detailed in the fourth report. At the end of the war, the NG&SF team still did not know if Bacinol was actually penicillin until they tested it against some penicillin from England, proving it to be the same compound. NG&SF began marketing the penicillin they produced in January 1946. Although the original building where Bacinol was produced was demolished, NG&SF named a new building in honor of their WWII efforts (Figure). 

The Nazis eventually succeeded in making penicillin by October 1944. However, Allied air raids crippled mass production of the drug (*9*).

## Patents

The issue of a patent for penicillin was a controversial problem from the beginning. Chain believed that obtaining a patent was essential. Florey and others viewed patents as unethical for such a life-saving drug. Indeed, penicillin challenged the basic notion of a patent, considering it was a natural product produced by another living microorganism. The prevailing view Great Britain at the time was that a process could be patented, but the chemical could not. Merck (New York, NY, USA) and Andrew Jackson Moyer each filed patents on the process of penicillin production with no opposition. Eventually, at war’s end, British scientists were faced with paying royalties for a discovery made in England. The penicillin production at NG&SF turned out to be more than of historical interest. Because NG&SF had researched and developed their own penicillin using their own mold culture, *P. baculatum*, and used their own production methods, they were not embroiled in any patent clash; the marketing of their penicillin eventually increased penicillin supply and decreased prices.

## Nobel Prize in 1945

Penicillin’s colossal effects led to the awarding of the Nobel Prize in Medicine and Physiology in 1945 to Fleming, Chain, and Florey. Penicillin was isolated from other microorganisms, which led to a new term, antibiotics. Using similar discovery and production techniques, researchers discovered many other antibiotics in the 1940s and 1950s: streptomycin, chloramphenicol, erythromycin, vancomycin, and others.

## Conclusions

Lessons can be learned from the circumstances surrounding the discovery of penicillin. The US government’s successful takeover of penicillin’s production and the unprecedented cooperation among drug companies (and nations) should strongly encourage public/private partnerships as we search for additional effective antimicrobial drugs. In addition, despite their essential value in modern medicine, antibiotics are also the only class of drugs that lose their efficacy with large-scale use as bacteria develop antibiotic resistance. We now are struggling with resistant bacteria that cause infections that are virtually untreatable. Infections such as those occurring after transplantation and surgical procedures, caused by these highly antibiotic-resistant pathogens, are threatening all progress in medicine. Yet, drug companies, some of the same companies that helped develop penicillin, have nearly abandoned efforts to discover new antibiotics, finding them no longer economically worthwhile. The dry pipeline for new antibiotics has led the Infectious Diseases Society of America and others to call for a global commitment to the development of new agents (*10*). We also must expertly manage the drugs that are currently available. The noteworthy serendipity involved in the discovery of penicillin should remind us that new antibiotics are difficult to find and, more important, should make us mindful when using these limited medical treasures.
